# Molecular Epidemiology of Peste des Petits Ruminants Virus Circulating in Fat‐Tailed Sheep in Bangladesh

**DOI:** 10.1002/vms3.71132

**Published:** 2026-08-07

**Authors:** Md. Arif Khan, Pronesh Dutta, Md. Kaisar Rahman, Abdullah‐Al Mamun, Jade K. Forwood, Shariful Islam, Ariful Islam

**Affiliations:** ^1^ Zoonotic Disease Research Program Institute of Epidemiology, Disease Control and Research (IEDCR), Mohakhali Dhaka Bangladesh; ^2^ Gulbali Research Institute Charles Sturt University Wagga Wagga New South Wales Australia

**Keywords:** fat‐tailed sheep, Peste des petits ruminants (PPR), phylogenetic analysis, ruminants, transboundary, vaccine

## Abstract

**Background:**

Peste des petits ruminants (PPR) is a highly contagious viral disease of sheep and goats that causes substantial economic losses through high morbidity, mortality and rapid transmission among small ruminants. Molecular epidemiological data on circulating PPR virus (PPRV) strains in Bangladesh remain limited.

**Objectives:**

To investigate the molecular epidemiology and phylogenetic characteristics of PPRV among fat‐tailed sheep and goats traded in livestock markets in Bangladesh.

**Methods:**

A cross‐sectional survey was conducted in livestock markets in Dhaka city, Bangladesh, during the Eid‐ul‐Adha festival between August and October from 2015 to 2018. Oral, nasal and rectal swabs were collected from 161 animals comprising 141 sheep (including 111 fat‐tailed sheep) and 20 goats. Samples were screened for PPRV using conventional hemi‐nested polymerase chain reaction (PCR) targeting the nucleocapsid gene. Positive amplicons were sequenced, analysed phylogenetically and subjected to three‐dimensional (3D) structural prediction of the RNA‐dependent RNA polymerase protein.

**Results:**

The overall prevalence of PPRV in small ruminants was 2.5% (95% confidence interval [CI]: 0.7–6.2). In fat‐tailed sheep, the prevalence was 3.6% (95% CI: 1.0–8.9), whereas no positive cases were detected among goats. A significantly higher (*p* < 0.05) prevalence was observed in animals sampled from the Gabtoli livestock market. Phylogenetic analysis revealed that two of the four positive samples clustered closely with PPRV strains from India and the United Arab Emirates, whereas the other two clustered with strains from China and Tibet.

**Conclusions:**

These findings demonstrate the transboundary circulation of genetically diverse PPRV strains among small ruminants traded in livestock markets in Bangladesh. Strengthened molecular surveillance in livestock markets and coordinated regional control strategies are needed to support PPR prevention and eradication.

## Graphical abstract

This study reports the molecular detection and genetic characterization of peste des petits ruminants virus in fat‐tailed sheep in Bangladesh. Phylogenetic analysis reveals transboundary lineage circulation, highlighting silent virus movement during high‐risk livestock trading periods and the need for strengthened regional surveillance.

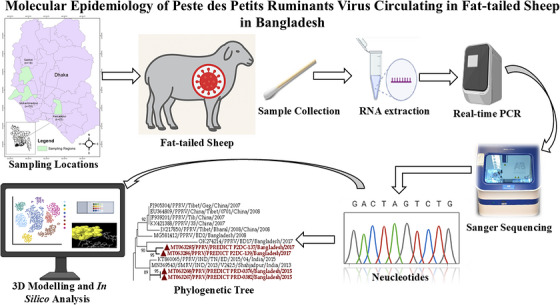

## 1 Introduction

*Peste des petits ruminants* (PPR), also known as small ruminant morbillivirus, is an acute and highly contagious viral disease of sheep and goats. The causative agent, PPR virus (PPRV), belongs to the genus *Morbillivirus* within the family Paramyxoviridae. The disease is characterized by high morbidity and mortality and poses a significant threat to the livestock sector due to frequent outbreaks and substantial economic losses. PPRV is evolutionarily related to other *Morbillivirus* members, including measles virus (MV), canine distemper virus (CDV), and rinderpest virus (RPV) (Barrett et al. 2006; Diallo 2006).

PPRV carries a single‐stranded RNA genome that encodes for six structural proteins: a nucleocapsid protein (N), a viral RNA‐dependent polymerase (L), an RNA‐polymerase phosphoprotein co‐factor (P), a matrix protein (M), a fusion protein (F), and a hemagglutinin protein (H) and two non‐structural proteins (C and V proteins) due to RNA editing of the phosphoprotein gene (Bailey et al. 2005). The N and F protein genes have been used extensively for genetic evolutionary analysis. Although only one serotype of PPRV is known to exist, genetically, PPRV strains are divided into four distinct lineages on the basis of partial sequences of the *N* and *F* genes (Banyard et al. 2010; Shaila et al. 1996). Geographically, Lineages I and II are mainly restricted to western and central Africa, Lineage III to eastern Africa and the Arabian Peninsula, and Lineage IV to South Asia, the Middle East, and, more recently, to Africa (Clarke et al. 2018; Dundon et al. 2020; Rahman et al. 2018; Shaila et al. 1996). In the South Asian subcontinent, Lineage IV PPRV was first reported in 1987 in the southern part of India and then in Pakistan in 1994 (Amjad et al. 1996) and Nepal in 1995 (Dhar et al. 2002).

In Bangladesh, PPRV was first identified by Islam et al. at the Institute of Animal Health, UK, now known as The Pirbright Institute, during an outbreak a long time back in 1993 (Amjad et al. 1996; Islam et al. 2001). Since then, this virus has become endemic and is now considered one of the major threats to the small ruminant population of Bangladesh. The disease can result in morbidity rates approaching 100% and mortality rates ranging from 23% to 100% in susceptible populations (Chowdhury et al. 2014). The overall prevalence of PPRV in Bangladesh was 31% in sheep and goats (Ahaduzzaman 2020). The disease imposes an economic burden, particularly on poor rural households dependent on small ruminants for livelihood and food security (Kotchofa et al. 2021). The estimated annual worldwide impact of PPR ranges from $1.4 to $2.1 billion (WOAH 2016).

Previously, few molecular studies were conducted on PPRV, mostly on seroprevalence (Razzaque et al. 2004), pathological diagnosis (Chowdhury et al. 2014), epidemiology (Rahman et al. 2018), vaccine and antibiotic combined immunotherapy (Islam et al. 2003). A limited number of studies described laboratory‐confirmed PPRV in goats and information about the genotypic diversity of PPRV in Bangladesh (Chowdhury et al. 2014; Rahman et al. 2016). In Bangladesh, small ruminants—primarily sheep and goats—have an estimated population of 31 million, accounting for 53% of the total ruminant population in the country (DLS 2025). Through the production of meat and dairy products, goats and sheep provide an essential source of nutrition and assist households, especially those in poverty, with food and stability (Prank et al. 2023). Though fat‐tailed sheep are increasingly becoming popular in Bangladesh, especially during Eid‐ul‐Adha, farmers and experts are not well‐versed in this developing type of livestock and how they might contribute to the Bangladeshi economy (Islam et al. 2018). Overall, 25% of the world's sheep population comprises fat‐tailed breeds (Mohapatra and Shinde 2018) that can adapt to the extreme environmental conditions and are a valuable energy reservoir for them in migration and the winter season (Moradi et al. 2012).

There are currently no published reports on PPRV infection in fat‐tailed sheep in Bangladesh. This study is the first to investigate the circulation of PPRV in this population, with a focus on molecular epidemiology and phylogenetic characterization.

## 2 Methods

### 2.1 Ethical Approval

Animal capture and handling were carried out in accordance with established protocols for animal safety and ethics by trained wildlife veterinarians, ensuring both animal welfare and the safety of all personnel involved in the research programme.

### 2.2 Study Location and Duration

**FIGURE 1:**
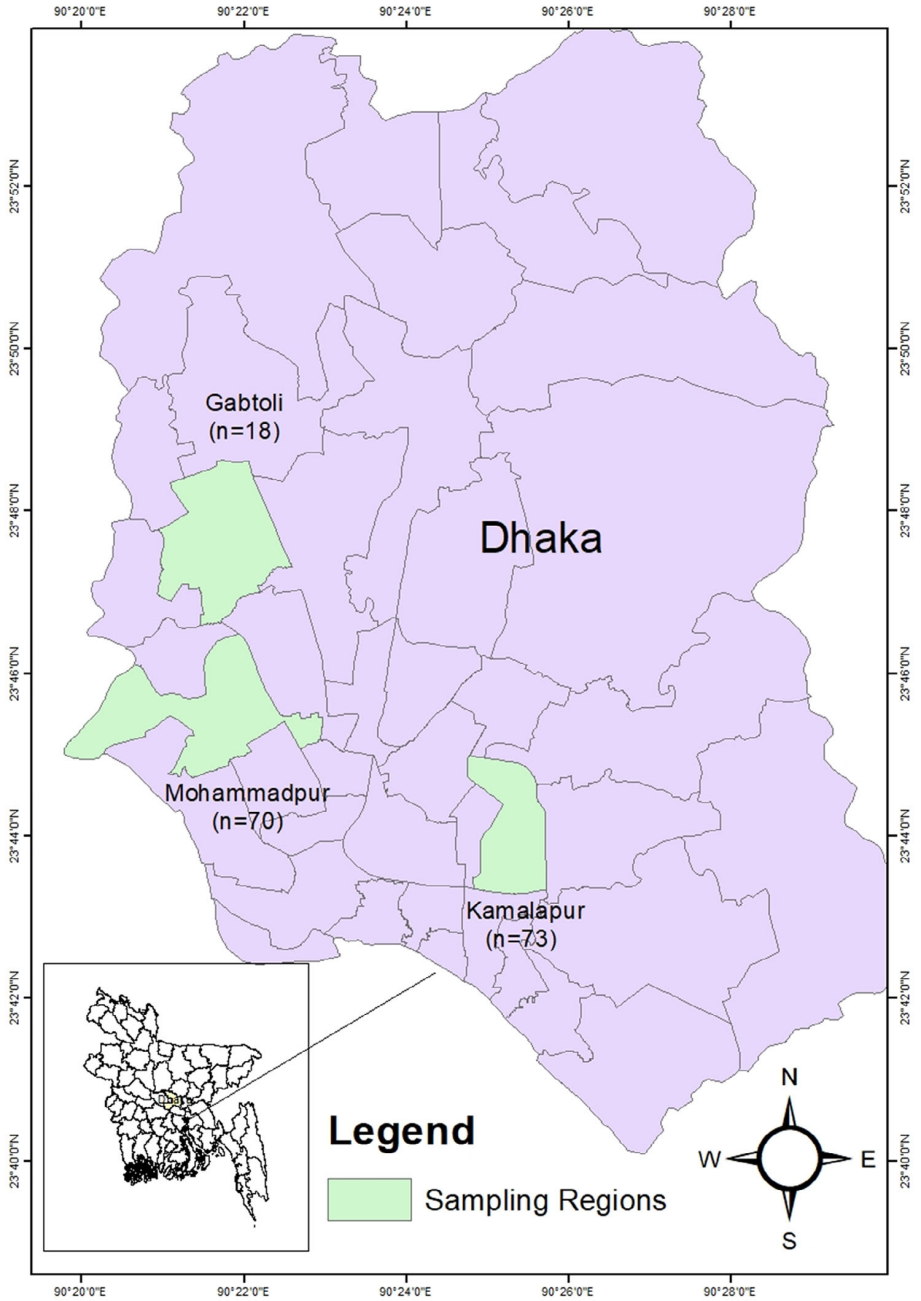
Sampling sites of the present study are graphically presented. The map was prepared with ArcGIS.v10.4.1 (ESRI, Redlands, CA, USA) using a shapefile from DIVA‐GIS (https://www.diva‐gis.org/). Here, *n* denotes the number of samples collected from each specific location of Gabtoli, Kamalapur and Mohammadpur.

A cross‐sectional study was conducted at three different locations as follows: Gabtoli, Mohammadpur, and Motijheel in Dhaka city, Bangladesh, from August to October over three consecutive years from 2015 to 2018 during the religious festival (Figure 1). In Bangladesh, those three locations are the primary source of imported animals for sale during Eid‐ul‐Adha. Gabtoli, the largest livestock market in Bangladesh, where imported fat‐tailed sheep and goats were brought for sale during the festival. The Mohammadpur and Motijheel sites are the stocked farms where importers keep different imported animals (e.g., dromedary camels and fat‐tailed sheep) with their regular raised local animals.

### 2.3 Sample Collection and Processing

Between 2015 and 2018, a total of 161small ruminants, including goats (n = 20) and sheep (n = 141, including 111 imported fat‐tailed sheep) were available in the study locations, and biological samples were collected. From each animal, oropharyngeal, nasal, and rectal swabs were collected using sterile swab sticks. Swab samples were kept in cryovials containing viral transport medium. All the cryovials were stored in a dry shipper containing liquid nitrogen (−196°C) in the field site and later transferred the vials to a −80°C freezer laboratory until laboratory testing.

#### 2.3.1 Data Collection

We collected the dataset using a structured questionnaire by expert veterinarians. We captured detailed information at the individual level with face‐to‐face interactions with animal owners or caretakers. We recorded the individual animals’ demographic information, including species, variety, breed, age and sex. Additionally, we collected the data related to the health status of the animals, seasonal variation, and locations. Most of the sampled small ruminants were of exotic breeds; however, a few indigenous breeds were also included, as they were present at the same sampling locations. August was considered part of the wet season, whereas September and October were categorized as the dry season.

### 2.4 Molecular Detection of PPRV

We tested the samples for different paramyxoviruses. We used a conventional hemi‐nested polymerase chain reaction (PCR) assay as previously described by Tong et al. (2008) targeting the Polymerase (*pol*) gene against various paramyxoviruses. Tong et al. have conducted multiple alignments of viral genomes from 29 different Paramyxoviridae strains, along with *Morbillivirus*, and found that RNA‐dependent RNA polymerase coding sequences were the best conserved among other genes. Thus, the *L*‐gene was selected for broadly reacting PCR assays (Tong et al. 2008). This study was conducted as part of the USAID PREDICT‐2 project surveillance, which employs broad consensus PCR testing to detect all viruses within a particular family or genus and to potentially identify novel agents within that group. Similar study also found that the PCR assay based on the *L* ‐gene provides rapid and specific detection of PPRV from field samples of Indian isolates and others (Dinçer and Özkul 2015; Hemida et al. 2020; Muthuchelvan et al. 2005). RNAs were extracted from 100 µL of supernatant fluid of virus‐infected cells with the QIAamp Viral RNA Kit (Qiagen, Santa Clarita, CA, USA) according to the manufacturer's instructions. The RNA was eluted from the column in 50 µL of RNase‐free water. RNAs for the PPRV strains were obtained as extracts in TRIzol and prepared according to the instructions provided by the commercial source (Invitrogen, Carlsbad, CA, USA). Viral RNAs were going through amplification of the gene segments by using the following primers (Table 1), visualized via agarose gel electrophoresis. Amplicons from the final round of PCR were purified using the QIAquick PCR Purification Kit (Qiagen, Inc., Valencia, CA, USA). Subsequently, amplified and purified products of the expected PCR amplicon size went through sequencing according to the Sanger dideoxy sequencer. Moreover, we edited the sequences manually after sequencing in Geneious Pro (version 9.1.3, Biomatters, Auckland, New Zealand). All four *L*‐gene sequences of PPRV obtained in this study have been deposited in the GenBank database (available at https://www.ncbi.nlm.nih.gov/genbank/).

**TABLE 1:** Primers used in the detection of paramyxoviruses.

Target gene	Protocol	Sequences (5ʹ→3ʹ)	Amplicon size (bp)	Condition
Polymerase (*pol*) gene	Round 1	PAR‐F1: GAAGGITATTGTCAIAARNTNTGGAC	∼639	Round 1: 94°C for 5 min, followed by 40 cycles of 94°C for 1 min, 48°C for 1 min and 72°C for 1 min. Finish with a final extension of 72°C for 7 min
		PAR‐R: GCTGAAGTTACIGGITCICCDATRTTNC		
	Round 2	PAR‐F2: GTTGCTTCAATGGTTCARGGNGAYAA	∼561	Round 2: 94°C 2 min, then 40 cycles of 94°C for 30 s, 48°C for 30 s, 72°C for 30 s. Finish with 72°C for 7 min
		PAR‐R: Same Reverse primer as round 1		

### 2.5 Phylogenetic Analysis

In total four PPRV sequences detected from this study were submitted to GenBank. The sequences are available under accession numbers: MT063285, MT063286, MT063267, and MT063268. The nucleic acid sequences of the RNA‐dependent RNA polymerase (*L*) gene obtained from PCR products were aligned with other related sequences from the National Center for Biotechnology Information (NCBI) (https://www.ncbi.nlm.nih.gov). Reference nucleotide sequences were also accessed other than those found in this study, representing various locations, hosts, years, and clades of the viruses from NCBI for cross‐checking. For the maximum likelihood (ML) tree, phylogenetic analysis was conducted using IQ‐TREE v2.0 (http://www.iqtree.org) to infer the evolutionary relationships among the sequences. The phylogenetic tree was constructed using the ML method with the GTR + I + G model. For each tree, we used 1000 bootstrap replicates for generating the trees and visualized them using Figtree v1.4.4 (http://tree.bio.ed.ac.uk/software/figtree).

### 2.6 Statistical Analysis

We recorded the individual animals’ information, including the origin of the animals, breed, age, sex, and health status. The combined data from the field and laboratory were incorporated into the Excel‐365 (Microsoft Office 365, Redmond, WA, USA) spreadsheet for checking the errors and discrepancies before sorting, coding, and testing to ensure their integrity. Finally, the data were imported to STATA 18SE (StataCorp, College Station, TX, USA) for further statistical analyses. Univariable logistic regression analysis was performed to identify potential risk factors associated with PPRV infection. Variables with a *p* value <0.05 were considered statistically significant.

### 2.7 Homology Modelling and Structural Epitope Prediction

An exploratory approach was applied through in silico modeling and epitope mapping on the basis of partial *L‐* gene sequences to identify potential conserved regions of immunological interest. A three‐dimensional (3D) structure prediction of RNA‐dependent RNA polymerase proteins of Bangladeshi PPRVs was performed using the Phyre2 (Protein Homology/Analogy Recognition Engine) structure prediction server (http://www.sbg.bio.ic.ac.uk/phyre2/html/page.cgi?id=index), the most cited online protein fold identification server that uses a dataset of known proteins taken from different reliable databases (Kelley et al. 2015). The resulting 3D model was analysed and viewed in PyMOL version 3.0 viewer (https://www.pymol.org/). Different tools were employed for stereochemical analyses and model quality evaluation as per the instruction manual of the software (Laskowski et al. 1996; Xu and Zhang 2011). Structural predictions of epitopes were performed with the ElliPro antibody epitope prediction tool (http://tools.iedb.org/ellipro/) (Ponomarenko et al. 2008). Structural epitopes were generated from the PDB file of the PPRV L 3D protein model generated by RaptorX (Källberg et al. 2012). Epitope structure predictions were performed on the basis of default parameters (minimum score value 0.5 and maximum distance of 6 Å). High‐scoring 3D epitope structures were viewed with the Swiss‐Pdb viewer (Guex and Peitsch 1997).

## 3 Results and Discussion

The overall prevalence of PPR in small ruminants was 2.5% (*n* = 4; 95% confidence interval [CI]: 0.7–6.2), which is notably lower than the pooled prevalence of 15.7% reported in the comprehensive meta‐analysis of PPR in goats and sheep in Bangladesh between 2000 and 2019 by Hasib and Chowdhury (2020). However, the prevalence of the PPRV is reported as 60% in small ruminants globally (Mondal et al. 2024), and recently this prevalence is identified as 35.6% in sheep in Bangladesh (Rahman et al. 2023). A likely explanation for this discrepancy is the seasonal timing and context of our sampling, which occurred exclusively during the Eid‐ul‐Adha festival. During this time, livestock selected for sale and slaughter are typically healthy, high‐value animals, potentially skewing the results toward lower apparent prevalence rates, which is supported by the previous findings where the RNA prevalence of the PPRV was reported to be higher in Dhaka than in the other sampling areas (Rahman et al. 2023). In between the seasons, higher percentages of PPR were detected in the dry season (11.1%) than in the wet season (1.4%), which is identical to a previous study where the PPRV was detected higher in summer than in winter and rainy season in small ruminants (Rahman et al. 2018). This trend aligns with other studies that suggest climatic stress, animal congregation and market movements during the dry season can increase viral transmission (Dash et al. 2021). Within the sampled species, PPRV positivity was detected only in sheep (2.8%; 95% CI: 0.7–8.1), whereas no positive cases were detected in goats. Among the variety of small ruminants, all PPR‐positive cases were detected in fat‐tailed sheep (3.6%, 95% CI: 1–8.9), the high‐yielding sheep variety imported from the neighboring country. This finding supports the previous study where imported small ruminants were detected with high percentages of PPRV in Bangladesh (Rahman et al. 2023). Between the sexes, all PPR‐positive animals were female (4.3%, 95% CI: 1.2–10.5). We observed that all PPR‐positive animals were adult (2.8%, 95% CI: 0.8–6.9) and healthy (2.6%, 95% CI: 0.7–6.6). These findings suggest the potential for asymptomatic carriage of the virus, aligning with earlier studies indicating silent transmission among subclinically infected animals (Wasee Ullah et al. 2016).

A comparatively higher proportion of PPRV‐positive samples was observed in the Gabtoli livestock market (11.1%), followed by Mohammadpur (2.9%), whereas no positive cases were detected at the Motijheel site. No other variables in Table 2 showed statistically significant associations (Table 2). These findings indicated that the significantly higher positivity rate at the Gabtoli livestock market underscores the potential risk of disease introduction and spread through major animal trading hubs.

**TABLE 2:** Univariate association of peste des petits ruminants virus (PPRV) in fat‐tailed sheep with demographic and animal‐level factors.

Variable	Category	Percentage, (*n*)	95% CI	*p* value
Season	Dry (18)	11.1, (2)	1.4–34.7	0.13
	Wet (143)	1.4, (2)	0.2–4.9	
Location	Gabtoli (18)	11.1, (2)	1.4–34.7	0.024
	Mohammadpur (70)	2.9, (2)	0.3–9.9	
	Motijheel (73)	0	0–4.9	
Species	Goat (20)	0	0–16.8	0.446
	Sheep (141)	2.8, (4)	0.8–7.1	
Variety	Barbari (2)	0	0–84.2	0.968
	Fat‐tailed sheep (111)	3.6, (4)	1–8.9	
	Garol (22)	0	0–15.4	
	Jamnapari (5)	0	0–52.2	
	Indigenous goat (5)	0	0–52.2	
	Najdi (5)	0	0–52.2	
	Pashmina (8)	0	0–36.9	
	Indigenous sheep (3)	0	0–70.8	
Breed type	High‐yielding goat (15)	0	0–21.8	0.877
	High‐yielding sheep (138)	2.9, (4)	0.8–7.3	
	Indigenous goat (5)	0	0–52.2	
	Indigenous sheep (3)	0	0–70.8	
Sex	Female (94)	4.3, (4)	1.2–10.5	0.087
	Male (67)	0	0–5.4	
Age	Adult (144)	2.8, (4)	0.8–6.9	0.785
	Subadult (7)	0	0–41	
	Juvenile (10)	0	0–30.8	
Health status	Apparently healthy (153)	2.6, (4)	0.7–6.6	0.643
	Sick (8)	0	0–36.9	

**Nucleotide sequence accession numbers**: The partial four RNA‐dependent RNA polymerase gene sequences have been deposited in GenBank under accession numbers (MT063267, MT063268, MT063285 and MT063286).

### 3.1 Phylogenetic Analysis

**FIGURE 2:**
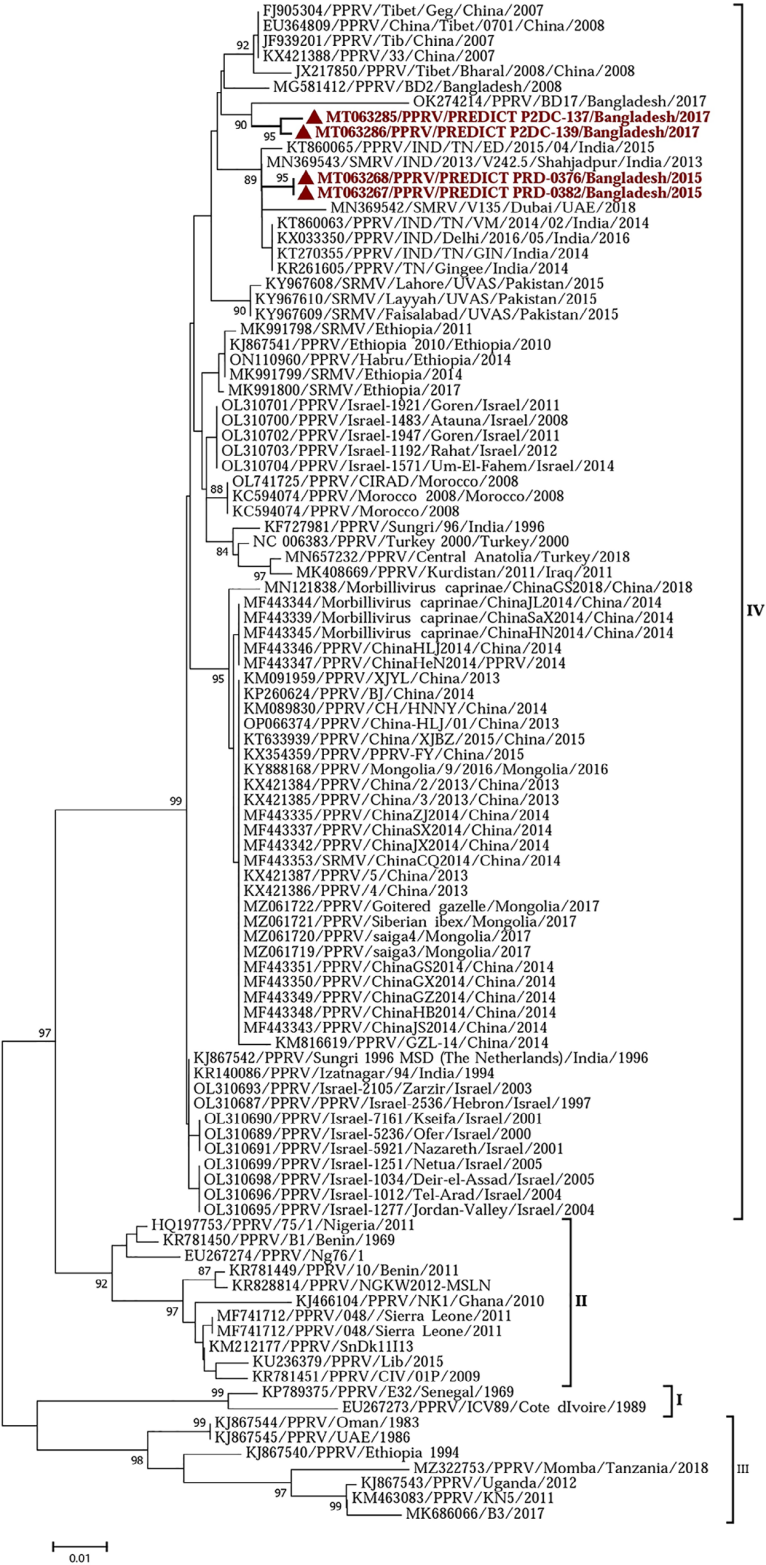
Phylogenetic analysis is based on the genome sequences of 4 PPRV RNA‐dependent RNA polymerase isolates highlighted as dark red (bootstrap 95%). Branch lengths are relatively short among the Bangladesh–India cluster. The topology shows four genetically distinct lineages (Lineage I–IV), consistent with the globally recognized PPRV classification. The analysis was based on 100 nucleotide sequences, and the scale bar (0.01) indicates minimal nucleotide divergence.

We conducted a comprehensive phylogenetic analysis of RNA‐dependent RNA polymerase gene sequences identified and collected from NCBI through BLAST analysis and found that the PPRV isolates belonged to Lineage IV (Figure 2). The percentages of replicate trees in which the associated taxa clustered together in the bootstrap analysis (1000 replicates) are shown next to the corresponding branches. The phylogenetic tree was constructed to scale, with branch lengths representing the same units as the evolutionary distances used to infer the tree. Evolutionary distances were estimated using the maximum composite likelihood method and are expressed as the number of base substitutions per site. The topology shows four genetically distinct lineages, consistent with the globally recognized PPRV classification. East of the lineage is supported by high bootstrap values (mostly >90%), which confirms the robust clustering. The majority of the tree—including all recent isolates from Bangladesh, India, China and the Middle East—belongs to Lineage IV. Similar phylogenetic and molecular observations were also found in different studies based on Bangladeshi PPRV sequences (Clarke et al. 2018; Nooruzzaman et al. 2021; Rahman et al. 2018, 2023). There were at least seven regional sub‐clusters found under Lineage IV across the East African country, Ethiopia and the North African country, Morocco, with different geographical locations in Asia. The ML tree also indicates that the viruses we detected in the fat‐tailed sheep host are like those found in goats.

Phylogenetic analysis of our four partial *L*‐gene sequences placed all Bangladeshi isolates within PPRV Lineage IV with strong bootstrap support (≥95%). Two of them are closely connected with another Bangladeshi PPR isolate (PPRV/Bangladesh/BD17/2017) reported by Nooruzzaman et al., which was isolated from goat in the same year of our study. All the four sequences are grouped in the same clade with another goat isolate from Bangladesh (PPRV/Bangladesh/BD2/2008). This strengthens and supports the phylogenetic analysis (Nooruzzaman et al. 2021). The isolates form a compact *L*‐gene subclade closely related to contemporary Indian sequences (2013–2016). Among the four virus sequences analyzed in this study, two were found to be closely clustered with PPRV strains previously identified in neighboring South Asian countries, including India and the United Arab Emirates (UAE), whereas the other two exhibited close genetic relationships with strains circulating in China and the Tibet Autonomous Region. This pattern of phylogenetic clustering highlights the complex and transboundary nature of PPRV transmission in the region. Similar findings have been reported in previous molecular epidemiological studies (Nooruzzaman et al. 2021; Rahman et al. 2023), reinforcing the notion that regional connectivity plays a critical role in the spread of the virus. One of the key factors contributing to this pattern is the porous nature of borders in South Asia and the high volume of both legal and illegal animal movement across these regions. The Indo‐Bangladesh border serves as a hotspot for cross‐border trade and livestock exchange, creating opportunities for the virus to spread silently between animal populations. Furthermore, the presence of a multi‐country corridor, spanning from Tibet through Nepal and India into Bangladesh, serves as a significant route for the transboundary movement of animals, both for commercial purposes and subsistence agriculture. These corridors not only facilitate the spread of livestock but also act as transmission hubs for infectious pathogens, including PPRV, implying epizootic continuity across the border (Clarke et al. 2018; Muthuchelvan et al. 2014). In short, the high sequence homology and short branch lengths indicate recent common ancestry, consistent with regional transmission and endemic maintenance of Lineage IV PPRV in South Asia.

### 3.2 Homology Modelling and Structural Epitope Prediction

**FIGURE 3:**
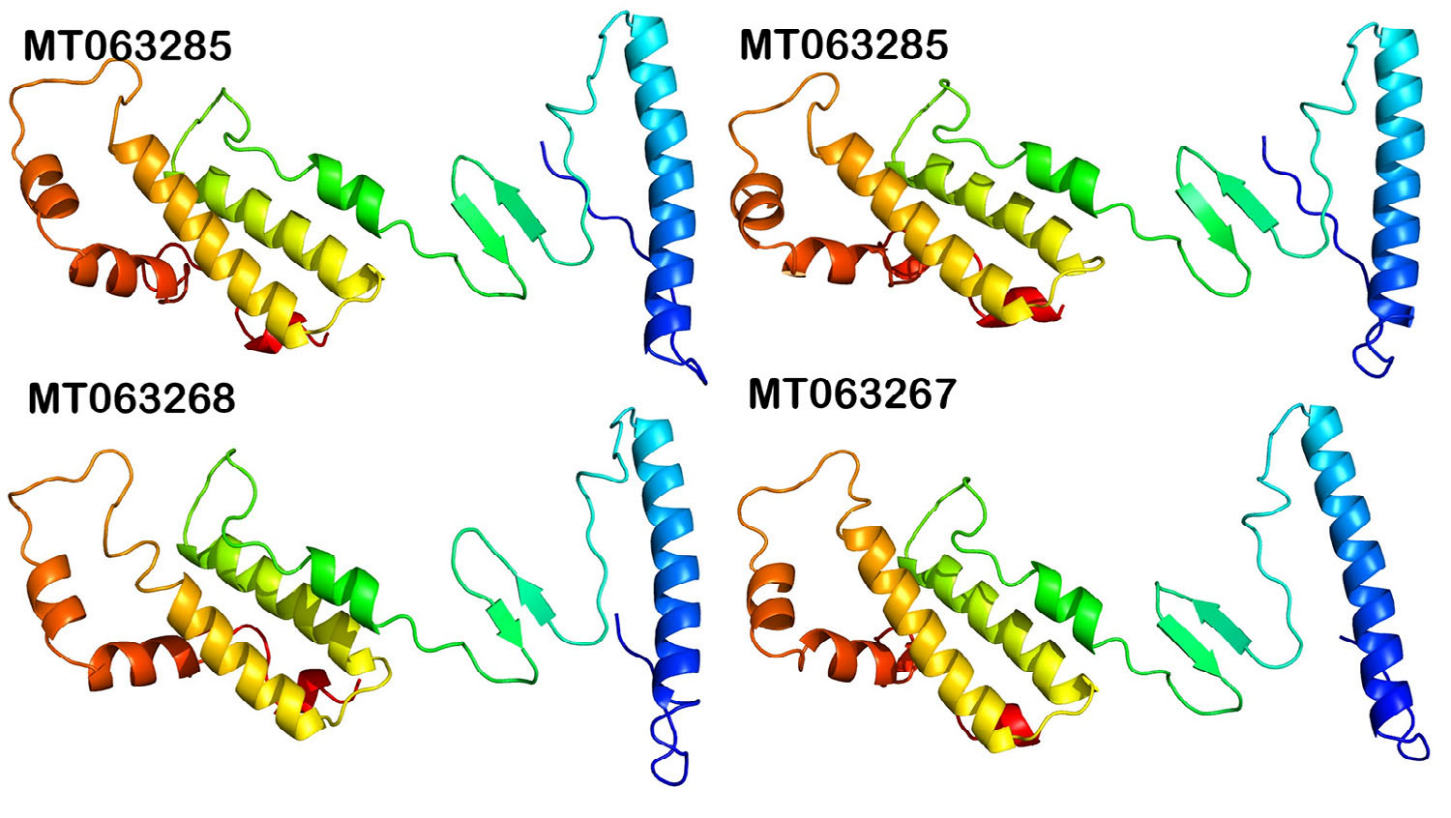
Three‐dimensional (3D) structure of RNA‐dependent RNA polymerase proteins (L) of PPRV of Bangladeshi strains (MT063285, MT063286, MT063267 and MT063268) generated by Phyre2 structure prediction server (http://www.sbg.bio.ic.ac.uk/phyre2/html/page.cgi?id=index) with intensive modelling mode and later analysed and visualized in PyMOL version 3.0 viewer (https://www.pymol.org/).

The 3D structure of the partial four RNA‐dependent RNA polymerase protein sequences of the PPRV of Bangladeshi strain was performed using the Phyre2 structure prediction server and graphically presented in Figure 3. Overall, 100% of the residues were modeled at >90% confidence in the case of all the PPRV strains. On the basis of the 3D structures of four RNA‐dependent RNA polymerase protein sequences, ElliPro, the structure‐based tool, was used to predict linear epitopes of PPRV L protein. ElliPro employed three algorithms to approximate the protein shape as an ellipsoid and later calculate the residue protrusion index and finally cluster neighboring residues based on their PI values (Ponomarenko et al. 2008).

The usefulness of employing prediction tools to assess the effectiveness of putative epitope binding has been demonstrated by numerous in silico investigations (Hasan et al. 2015; Khan et al. 2015; Mahfuz et al. 2021; Nooruzzaman et al. 2021; Wang et al. 2016). A total of eight linear and eight discontinuous epitope peptides were predicted on the basis of the score (Table 3). We identified three major conserved antigenic regions across all four L protein sequences (MT063285, MT063286, MT063268 and MT063267), corresponding to N‐terminal, central and C‐terminal peptide clusters. We observed the primary motifs, KRVPSSWPYSL, VLRQRLHDVGHHL(K/A)ANE and INTTMT(Q/D)—these were consistently detected in both linear and discontinuous predictions with high ElliPro scores (0.80–0.90). This convergence indicates that these antigenic regions are structurally stable and potentially immunological epitopes suitable for downstream peptide‐based diagnostic or vaccine‐focused analyses.

**TABLE 3:** ElliPro predicted linear and discontinuous epitope peptides of peste des petits ruminants virus (PPRV) RNA‐dependent RNA polymerase protein, showing conserved antigenic motifs across Bangladeshi strains (MT063285, MT063286, MT063267 and MT063268).

Protein ID	Start	End	Sequence (peptides)	Length	Score
**MT063285**	32	46	VLRQRLHDVGHHLKA	15	0.884
	7	17	KRVPSSWPYSL	11	0.882
	131	141	NFTINTTMTQD	11	0.817
MT063286	7	17	KRVPSSWPYSL	11	0.907
	32	48	VLRQRLHDVGHHLKANE	17	0.884
MT063268	3	13	KRVPSSWPYSL	11	0.891
	28	44	VLRQRLHDVGHHLKANE	17	0.892
	130	136	INTTMTQ	7	0.85
MT063267	26	39	VVLRQRLHDVGHHL	14	0.901
	128	135	TINTTMTQ	8	0.844
	1	15	TKRVPSSWPYSLKKR	15	0.841
Discontinuous					
**MT063285**	_:T6, _:K7, _:R8, _:V9, _:P10, _:S11, _:S12, _:W13, _:P14, _:Y15, _:S16, _:L17, _:R20				0.859
	_:V32, _:R34, _:Q35, _:R36, _:L37, _:H38, _:D39, _:V40, _:G41, _:H42, _:H43, _:L44, _:K45, _:A46, _:N47, _:E48, _:T49				0.843
	_:N131, _:F132, _:T133, _:I134, _:N135, _:T136, _:T137, _:M138, _:T139, _:Q140, _:D141				0.817
MT063286	_:T6, _:K7, _:R8, _:V9, _:P10, _:S11, _:S12, _:W13, _:P14, _:Y15, _:S16, _:L17, _:R20				0.878
	_:V32, _:R34, _:Q35, _:R36, _:L37, _:H38, _:D39, _:V40, _:G41, _:H42, _:H43, _:L44, _:K45, _:A46, _:N47, _:E48, _:T49, _:I50				0.846
MT063268	_:V28, _:R30, _:Q31, _:R32, _:L33, _:H34, _:D35, _:V36, _:G37, _:H38, _:H39, _:L40, _:K41, _:A42, _:N43, _:E44, _:T45				0.878
	_:T2, _:K3, _:R4, _:V5, _:P6, _:S7, _:S8, _:W9, _:P10, _:S12, _:L13, _:R16				0.872
	_:F128, _:T129, _:I130, _:N131, _:T132, _:T133, _:M134, _:T135, _:Q136, _:D137				0.814
MT063267	_:V26, _:V27, _:R29, _:Q30, _:R31, _:L32, _:H33, _:D34, _:V35, _:G36, _:H37, _:H38, _:L39, _:A41				0.895
	_:T1, _:K2, _:R3, _:V4, _:P5, _:S6, _:S7, _:W8, _:P9, _:Y10, _:S11, _:L12, _:K13, _:R15				0.855
	_:F127, _:T128, _:I129, _:N130, _:T131, _:T132, _:M133, _:T134, _:Q135, _:D136				0.802

**FIGURE 4:**
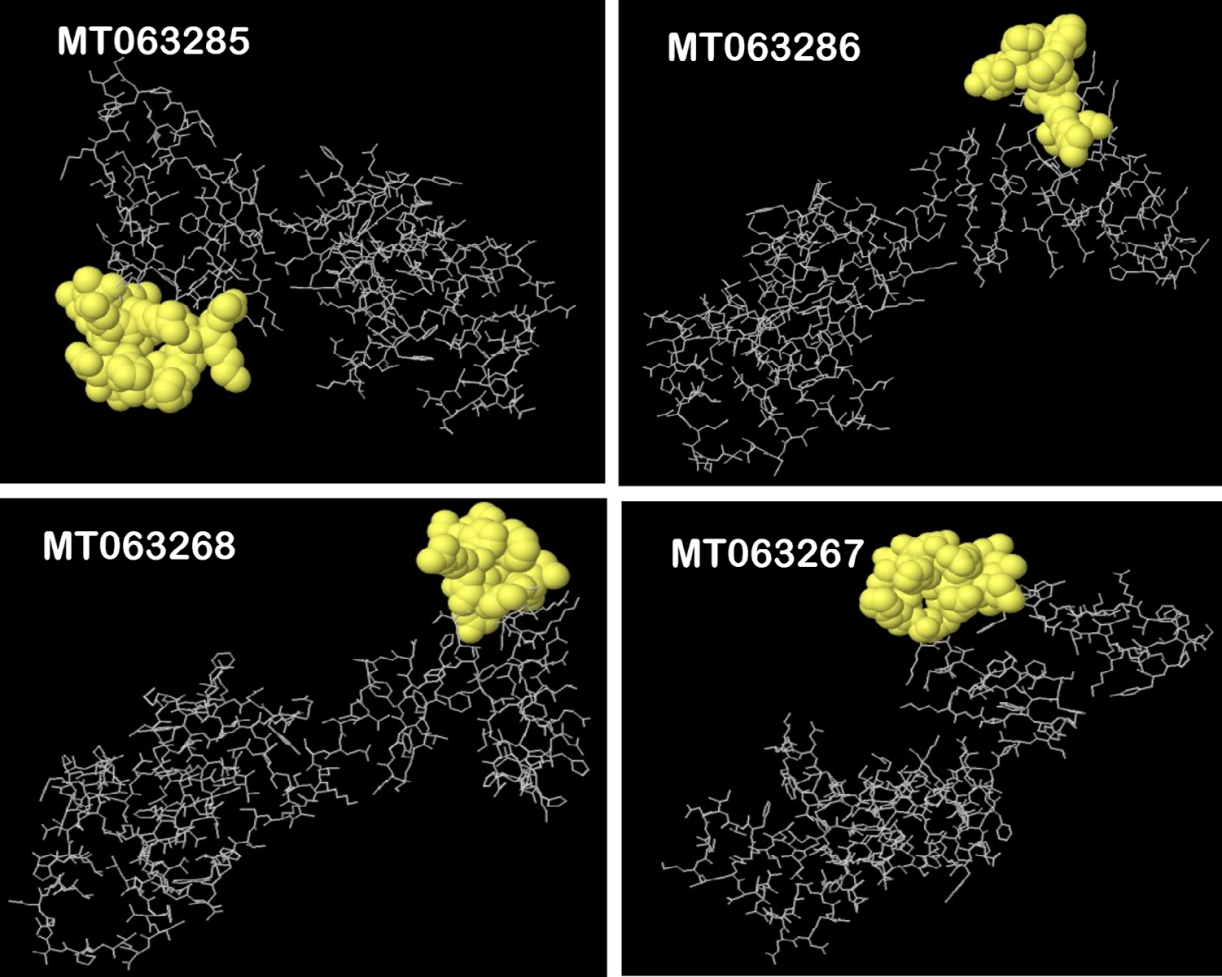
Antibody epitopes with the highest score in the 3D structure of the Bangladeshi PPRV RNA‐dependent RNA polymerase proteins (MT063285, MT063286, MT063267 and MT063268) predicted by ElliPro (http://tools.iedb.org/ellipro/). Here, predicted linear epitopes are highlighted in yellow space‐filling (spherical) models representing amino acid residues, while the overall backbone protein structures are displayed in wireframe format (grey colour).

The prediction of the antibody epitopes with the highest score in the 3D structure of the Bangladeshi PPRV RNA‐dependent RNA polymerase proteins was performed using the ElliPro antibody epitope prediction tool (Ponomarenko et al. 2008) and shown in Figure 4.

These high‐scoring predicted epitopes are selected on the basis of their surface accessibility as well as their antigenic reactivity with immunoglobulins of humoural immunity (Vita et al. 2015). These identified epitopes may serve for the multi epitope vaccine development against PPRV in Bangladesh upon further in vivo and in vitro assessment for the confirmation of this study. These efforts might save time and cost for the future study to combat PPRV (Diallo 2006; Tomar and De 2010). Because of the limited sequence coverage and absence of experimental validation, these predictions were considered preliminary and hypothesis‐generating, rather than confirmed antigenic determinants against PPRV.

In our study we have some limitations. We were unable to include a statistically appropriate sample size, as we collected a disproportionate sample size of exotic animal species due to the unavailability of a representative number of exotic animals during the study period. The multivariable model in statistical analysis was unable to be conducted because of having an inappropriate sample size and low positive cases of PPRV. No PPRV was identified in goats in our study, which might be a consequence of sampling a few goats depending on the availability of the exotic goats in the markets during the study period in those specific animal markets.

Another limitation is that we were unable to examine other genes of PPRV except the *L*‐gene due to funding constraints. We believe that this study would have enhanced the PPRV research in fat‐tailed sheep in Bangladesh. Consequently, we have planned and suggested that additional studies should be carried out to assess the genetic diversity of PPRV isolates in fat‐tailed sheep through whole‐genome sequencing.

## 4 Conclusions

This study reports the molecular detection of PPRV in fat‐tailed sheep in Bangladesh, with a prevalence of 2.5%, where location was a significant risk factor, particularly in the Gabtoli livestock market. All positive cases were asymptomatic adult females of the fat‐tailed breed, indicating potential for silent transmission. Phylogenetic analysis confirmed that all strains belonged to Lineage IV, closely related to regional variants. Structural analysis suggested several conserved epitopes, providing preliminary insights that may assist future diagnostic or vaccine development research. These in silico findings are exploratory and hypothesis‐generating, intended to guide subsequent experimental validation of PPRV polymerase immunogenicity rather than to propose confirmed vaccine targets. These findings emphasize the importance of transboundary disease surveillance focused on control strategies and molecular epidemiology to prevent and control PPRV in exotic small ruminant populations, especially during high‐risk events like religious festivals.

## Author Contributions

**Md. Arif Khan**: writing – original draft, formal analysis, methodology, data curation, validation software, visualization. **Pronesh Dutta**: data curation, validation, formal analysis, writing – review and editing. **Md. Kaisar Rahman**: investigation, methodology, data curation, writing – review and editing. **Abdullah‐Al Mamun**: investigation, data curation, writing – review and editing. **Jade K. Forwood**: writing – review and editing, resources, funding acquisition. **Shariful Islam**: investigation, methodology, data curation, validation, writing – review and editing. **Ariful Islam**: conceptualization, supervision, project administration, writing – review and editing, resources, methodology, funding acquisition.

## Funding

This study was supported by the United States Agency for International Development (USAID) under the Emerging Pandemic Threats PREDICT project (Cooperative Agreement No. AID‐OAA‐A‐14‐00102).

## Ethics Statement

The animal study was reviewed and approved by the Animal Experimental Ethics Committee (AEEC) of Chattogram Veterinary and Animal Sciences University (CVASU), Chattogram, Bangladesh. The AEEC‐CVASU permit number of this study was (CVASU/Dir (R&E) AEEC/2015/751, dated: 05/09/2016).

## Conflicts of Interest

The authors declare no conflicts of interest.

## Data Availability

The data generated and analysed during this study are included in the article. The nucleotide sequences are publicly available in GenBank. Additional raw datasets are available from the corresponding author upon reasonable request.
